# Curcumin exerts protective effects against valproic acid-induced testicular damage through modulating the JAK1/STAT–3/IL–6 signaling pathway in rats

**DOI:** 10.22038/ijbms.2024.76948.16659

**Published:** 2025

**Authors:** Eda Dokumacioglu, Hatice Iskender, Kubra Asena Terim Kapakin, İSmail Bolat, Behzat Mokhtare, Ali Dogan Omur, Armagan Hayirli

**Affiliations:** 1 Eda Dokumacioglu, Department of Nutrition and Dietetics, Faculty of Health Sciences, Artvin Coruh University, Artvin 08000, Turkey; 2 Department of Pathology, Faculty of Veterinary Medicine, Ataturk University, Erzurum 25240, Turkey; 3 Department of Pathology, Faculty of Veterinary Medicine, Dicle University, Diyarbakır, 21000, Turkey; 4 Department of Reproduction and Artificial Insemination, Faculty of Veterinary Medicine, Ataturk University, Erzurum 25240, Turkey; 5 Department of Animal Nutrition and Nutritional Disorders, Faculty of Veterinary Medicine, Ataturk University, Erzurum 25240, Turkey

**Keywords:** Curcumin, Interleukin–6, Janus kinases, Testicular damage, Valproic acid

## Abstract

**Objective(s):**

This experiment was carried out to investigate the protective effects of curcumin (CUR) on testicular damage induced by the valproic acid (VPA) administration.

**Materials and Methods:**

Male Wistar–Albino rats (n=28, 250–300 g) were randomly divided into four groups: Control (1 ml saline, oral), VPA (500 mg/kg, IP), CUR (200 mg/kg, oral), or VPA+CUR (500 mg/kg, VPA, IP plus 200 mg/kg CUR, oral). The treatments were applied for 14 days. Serum testosterone and testis [Janus kinases1 (JAK1), signal transducers and activators of transcription–3 (STAT–3), interleukin–6 (IL–6), malondialdehyde (MDA), tumor necrosis factor-alpha (TNF–α), interleukin–18 (IL–18), and nuclear factor (NF)–κB)] samples were collected for biochemical analyses. Semen samples were subjected to microscopy for spermatological parameters. Testis tissue was also analyzed for histopathological and immunohistochemical methods.

**Results:**

The VPA administration caused a 37% decrease in serum testosterone concentration and 5.32, 9.51, 2.44, and 3.68–fold increases in testicular tissue JAK1, STAT–3, IL–6, and MDA levels, respectively. There were also 50, 52, and 72% reductions in sperm motility, sperm viability, and the mean testicular biopsy score, respectively, accompanied by considerable degenerative changes and necrosis in seminiferous tubules in the VPA group. There is also an immune-positive reaction for IL–18 and NF–κB in only Leydig cells.

**Conclusion:**

The CUR treatment may be beneficial in restoring testicular damage through antiinflammatory and anti-oxidant potential.

## Introduction

Valproic acid (VPA) is an antiepileptic and mood stabilizer drug that is commonly used in different psychiatric disorders, including bipolar disorder, post-traumatic stress disorder, treatment-resistant depression, and treatment-resistant schizophrenia, as well as some neurological conditions, including epilepsy, neuropathic pain, tremor, and migraine prophylaxis (1). Although VPA is a very safe drug with wide therapeutic properties, it may exert side effects, such as nausea, vomiting, gastrointestinal, pancreatitis, hematological, hormonal, bleeding, hypotension, tachycardia, respiratory failure, decreased serum carnitine level, and disturbances in lipid, carbohydrate, and urea metabolism (2, 3). Moreover, VPA administration causes atrophy of the testis, epididymis, prostate gland, and seminal vesicles (4), which results in decreased libido and sex hormone levels. The molecular mechanism underlying the adverse effects of VPA on the male reproductive system remains to be elucidated (5).

Considerable proof supports the role of signal transducers and activators of transcription–3 (STAT–3) in the arrangement of apoptosis. STAT–3 belongs to a family of transcription factors activated by Janus kinases (JAK) through phosphorylation of tyrosine705 in response to various cytokines (6, 7). The JAK/STAT signaling pathway is an important cellular signal transduction pathway regulating various cellular physiological processes, including proliferation, differentiation, apoptosis, and death. The JAK1/STAT–3 signaling pathway is activated to regulate the expression of inflammatory factors such as interleukin–6 (IL–6). This may eventually aggravate inflammation and is considered an important therapeutic target for novel drug development (8). Tumor necrosis factor–alpha (TNF–α) is a major mediator of inflammation regulated by the activation of nuclear factor (NF)–κB, a transcription factor. Additionally, most inflammatory cytokines also activate TNF–α and NF–κB. Consequently, agents that downregulate NF–κB levels may influence these diseases (9).

Curcumin (CUR), the main ingredient in turmeric, is one of the most popular phytochemicals (10). It is commonly used in traditional medicine thanks to its antiinflammatory (11), anti–carcinogenesis (12), anti-oxidant (13), and hypocholesterolemic (14) properties. The beneficial effects of supplemental CUR were shown to promote permeability of pancreatitis, gout, inflammatory bowel disease, colorectal cancer, and hepatic fibrosis through various mechanisms, notably by suppressing inflammation (15,16). CUR is capable of suppressing both acute and chronic inflammation. The antiinflammatory mechanism of CUR involves attenuating the inflammatory response in TNF-α stimulated human endothelial cells by interfering with the NF-κB signaling pathway (17). CUR possesses a powerful ability to neutralize superoxide radicals, hydrogen peroxide, and nitric oxide (NO) generated by activated macrophages. It also plays a role in reducing iron complexes and inhibiting lipid peroxidation. CUR effectively scavenges various reactive oxygen species produced by macrophages, including superoxide anions, hydrogen peroxide, and nitrite radicals. These actions are likely key mechanisms through which CUR exerts its anti-oxidant effects (18). This experiment was conducted to determine if CUR has therapeutic effects on testicular damage induced by VPA administration in rats.

## Materials and Methods

***Experimental design***

Atatürk University Local Ethics Committee for Animal Experiments approved this experimental protocol (2021/56). A total of 28 male Wistar–Albino rats, weighing 250–300 g, were kept at standard housing facilities (21–22°C, 60±5% humidity, and 12 hr light:12 hr dark cycle) and fed a standard laboratory chow diet and water *ad libitum*.

 The rats were randomly divided into four groups: 1 ml of saline via oral gavage (Control), 500 mg/kg VPA (dissolved in distilled water) by intraperitoneally (5) (VPA), 200 mg/kg CUR via oral gavage (19) (CUR), or 500 mg/kg VPA plus 200 mg/kg CUR (VPA+CUR). The treatments were applied for 14 days. The VPA (catalog no: 1069–66–5) and CUR (catalog no: 458–37–7) were purchased from Sigma Aldrich (Sigma Chemical Co., St. Louis, MO, USA).

Blood and testis samples were collected for biochemical analyses and histopathological evaluations. Prior to blood sampling intracardially, rats were administered ketamine (80 mg/kg; Ketalar®, 50 mg/ml, Eczacibasi, Istanbul, Turkey) and xylazine (10 mg/kg; Rompun®, 2%, Bayer, Istanbul, Turkey) at the end of the experiment. Then, they were sacrificed. After opening the abdominal wall, the testicles were exposed and removed.

***Biochemical analysis***

 Serum testosterone (SunRed, Biological Technology Co. Ltd, Shanghai, China) and testis tissue STAT–3 (Biocompare, South San Francisco, CA, USA), JAK1 (Biocompare), and IL–6 (SunRed) levels were measured by the Sandwich-ELISA method, based on a specific antigen and antibody reaction according to the manufacturer’s protocol. An enzyme is used as a marker to prepare the labeled conjugate. After completion of the reaction, separation was achieved by adding a substrate to the medium, and enzyme activity was measured spectrophotometrically. 

Testis tissue malondialdehyde (MDA) levels were measured based on a reaction with thiobarbituric acid at 90–95°C to yield a pink-colored chromogen (20). After 15 min, the absorbance values of the rapidly cooled samples were read spectrophotometrically at 532 nm. The MDA level was expressed as nmol/g tissue protein. Protein was assayed by the method of Bradford *et al*. (21), with serum bovine albumin as standard.

***Semen evaluation***

One cauda epididymis was used to obtain semen samples for each animal. Medley-selected cauda epididymidis was chopped in a Petri dish, including 5 ml of physiological saline. To provide the migrations of spermatozoa from cauda epididymidis to fluid, A 5–minute incubation period was acquired on the warmed stage at 35 °C. Then, following the incubation period, cauda epididymidis residue was eliminated using anatomical tweezers from the Petri dish. The fluid remaining in the Petri dish was used as a semen sample. Evaluation of semen was conducted using routine spermatological parameters, including motility and dead sperm rate (22). To evaluate the percentage of sperm motility, A light microscope (Primo Star; Carl Zeiss, Oberkochen, Germany) equipped with the heated stage was used to measure the percentage of sperm motility. Briefly, a slide was placed on a heated stage warmed up to 35 °C placed on a conventional light microscope. Approximately 20 ll of semen sample was dropped on the slide. The percentage of sperm motility was determined by visual examination of the sample. To evaluate sperm motility, randomly selected three areas from each sample were also assessed to predict sperm motility. The average of three field estimates ions was counted as the final motility score of the sample (23, 24).

 Determination of the dead sperm rate was observed under the light microscope. According to the staining of sperm heads, they were classified as dead (having a stained head) or live (having an unstained head) sperm cells. Randomly selected 300 sperm cells for each sample were investigated, and dead sperm rates counted as the percentage (25).

***Histopathological and immunohistochemical analysis***

Testes tissue samples were fixed with Bouin’s solution for 36 hr, dehydrated through a graded alcohol series, cleared with xylene, and embedded in paraffin wax. Sections were cut at 4 μm and mounted on slides. Sections were deparaffinized with xylene, rehydrated through graded alcohol solutions, and stained with hematoxylin and eosin (26).

Tissue sections were evaluated by high–power light microscopy (Olympus Bx51 with a DP72 camera system (Olympus Corp., Tokyo, Japan). Each specimen was examined in 10 randomly selected areas of approximately at 40x. The inflammation scores were graded as absent, mild, moderate, strong, and very strong if there was no staining (score 0), mild staining (score 1), moderate staining (score 2), strong staining (score 3), and very strong staining (score 4) (27).

The mean testicular biopsy score criteria were determined histopathologically (27). A score of 0–10 was given to each tubule according to epithelial maturation (1: No cells, 2: Sertoli cells without germ cells, 3: Only spermatogonia, 4: Only a few spermatocytes, 5: Many spermatocytes, 6: Only a few early spermatids, 7: Many early spermatids, 8: Few late spermatids, 9: Many late spermatids, and 10: Full spermatogenesis).

The sections from the testes samples (4 μm) were cut and prepared for immunohistochemical analysis of testicular cells by a standard avidin–biotin–peroxidase method (28). Briefly, rabbit polyclonal antibodies that react with rat TNF–α (catalog number: sc–52746651), IL–6 (catalog number: sc–57315), IL–18 (catalog number: sc–133127), and NF–κβ (catalog number: sc–8008) at the dilutions of 1:100, 1:100, 1:100, and 1:100, respectively were applied for 60 min (Santa Cruz Biotechnology, Dallas, US). An exposed mouse and rabbit-specific horseradish peroxidase/3,3–diaminobenzidine chromogen solution (HRP/DAB) estimation kit (ab80436; Abcam, Cambridge, UK). After three washes with 0.1% Tween 20 in phosphate-buffered saline, the sections were incubated with 3,3–diaminobenzidine (Dako Cytomation, Santa Clara, CA, USA) and counterstained with Mayer’s hematoxylin (Dako Cytomation). Each specimen was examined in 10 randomly selected areas of approximately at 40x. Immune positivity was graded as absent [no staining (score 0)], mild [mild staining (score 1)], moderate [moderate staining (score 2)], strong [strong staining (score 3)], and very strong [strong staining (score 4)] (29).

***Statistical analysis***

The continuous data were subjected to one–way ANOVA using the GLM Procedure (Statistical Analysis of the System, SAS, Version 9.0, SAS Institute Inc., Cary, NC, USA). The mean differences among the four groups were attained by the LSD option. The discrete data were subjected to the Wilcoxon rank sum test using the NPAR1WAY Procedure (SAS). Values for *P*≤0.05 were considered statistically significant.

**Table 1:** Effect of curcumin treatment on serum testosterone concentrations and testicular tissue transcription and antiinflammatory markers in rats exposed to valproic acid-induced testicular damage

	Parameters^1^				
Groups^2^	Testosterone (pg/ml)	JAK1 (ng/ml)	STAT–3 (ng/ml)	IL–6 (pg/ml)	MDA (nmol/g protein)
Control	80.2±0.4^a^	3.26±0.18^a^	1.81±0.04^c^	24.9±2.5^c^	5.95±0.70^c^
VPA	50.3±0.6^b^	17.3±2.4^b^	17.2±3.2^a^	60.7±6.2^a^	21.9±2.5^a^
CUR	70.9±0.6^a^	3.43±0.12^a^	1.86±0.07^c^	36.3±3.8^b^	9.57±0.74^b^
VPA+CUR	90.2±0.6^a^	6.60±1.33^a^	6.36±0.80^b^	37.6±1.3^b^	9.21±1.13^b^

Data are the least square means±SE. Different superscripts within columns differ (*P*<0.05).

^1^STAT–3 = Signal transducers and activators of transcription–3; JAK1 = Janus kinases, IL–6 = Interleukin–6, MDA = Malondialdehyde.

^2^Treatments were applied for 14 days. Control = rats given 1 ml of normal saline via oral gavage, VPA = 500 mg/kg valproic acid (dissolved in distilled water) intraperitoneally, CUR = 200 mg/kg curcumin via oral gavage, and VPA+CUR = 500 mg/kg VPA plus 200 mg/kg CUR.

^a^ Significantly different from the VPA group (*P*<0.05).

^b^ Significantly different compared to the control group (*P*<0.05).

^c ^Significantly different compared to the VPA+CUR group (*P*<0.05).

CUR: Curcumin; VPA: Valproic acid; JAK: Janus kinases; STAT-3: Transcription–3; MDA: Malondialdehyde

**Table 2:** Effect of curcumin treatment on sperm characteristics and testicular biopsy score in rats exposed to valproic acid-induced testicular damage

	Parameters		
Groups^1^	Motility (%)	Viable (%)	Testicular Biopsy Score^2^
Control	70.0±3.3^a^	57.0 ± 2.9^a^	8 (7–10)^a^
VPA	35.0±3.1^b^	27.3 ± 4.4^b^	2 (1–4)^d^
CUR	65.7±3.2^a^	23.9 ± 4.5^b^	7 (6–8)^b^
VPA+CUR	42.9±2.1^b^	34.3 ± 5.6^b^	5 (1–7)^c^

Data are the least square means±SE, except for testicular biopsy score [median (minimum–maximum]. Different superscripts within columns differ (*P*<0.05).

^1^Treatments were applied for 14 days. Control = rats given 1 ml of normal saline via oral gavage, VPA = 500 mg/kg valproic acid (dissolved in distilled water) intraperitoneally, CUR = 200 mg/kg curcumin via oral gavage, and VPA+CUR = 500 mg/kg VPA plus 200 mg/kg CUR.

^2^Based on Johnsen (1970). 1: No cells, 2: Sertoli cells without germ cells, 3: Only spermatogonia, 4: Only a few spermatocytes, 5: Many spermatocytes, 6: Only a few early spermatids, 7: Many early spermatids, 8: Few late spermatids, 9: Many late spermatids, and 10: Full spermatogenesis.

^a ^Significantly different from the VPA group (*P*<0.05).

^b ^Significantly different compared to the control group (*P*<0.05).

^c ^Significantly different compared to the VPA+CUR group (*P*<0.05).

^d ^Significantly different compared to the control group (*P*<0.05).

CUR: Curcumin; VPA: Valproic acid

**Figure 1:**
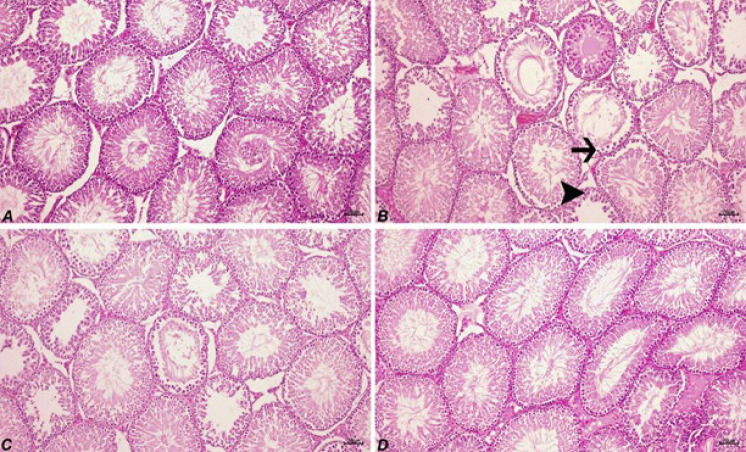
Degenerative (arrow) and necrotic (arrowhead) changes in seminiferous tubules epithelial cells, H&E, 70 µm

A: Normal appearance of testicular tissue in the control group. B: Intense in the VPA group, C: Mild degeneration in the CUR group, D: Moderate in the VPA+CUR group. Control = rats given 1 ml of normal saline via oral gavage, VPA = 500 mg/kg valproic acid (dissolved in distilled water) intraperitoneally, CUR = 200 mg/kg curcumin via oral gavage, and VPA+CUR = 500 mg/kg VPA plus 200 mg/kg CUR.

CUR: Curcumin; VPA: Valproic acid

**Table 3:** Effect of curcumin treatment on histopathology of seminiferous

	Parameters^2^	
Groups^1^	Degeneration	Necrosis	
Control	0 (0–1)^d^	0 (0–0)^c^	
VPA	4 (3–4)^a^	3 (2–3)^a^	
CUR	1 (1–2)^c^	0 (0–1)^c^	
VPA+CUR	3 (1–4)^b^	1 (0–2)^b^	

tubules epithelial cells on rats

Data are the median value (minimum-maximum). Different superscripts within columns differ

(*P*<0.05).

^1 ^The treatments were applied for 14 days. Control = rats given 1 ml of normal saline via oral gavage, VPA = 500 mg/kg valproic acid (dissolved in distilled water) intraperitoneally, CUR = 200 mg/kg curcumin via oral gavage, and VPA+CUR = 500 mg/kg VPA plus 200 mg/kg CUR.

^2^ Based on Apaydin–Yildirim et al. (2017). 0: No staining, 1: Mild staining, 2: Moderate staining, 3: Strong staining, and 4: Very strong staining.

^a ^Significantly different when compared with the control group (P<0.05).

^b ^Significantly different when compared with the VPA group (P<0.05).

^c ^Significantly different compared to the VPA+CUR group (*P*<0.05).

^d ^Significantly different from the CUR group (*P*<0.05).

CUR: Curcumin; VPA: Valproic acid

**Table 4:** Effect of curcumin treatment on immunohistochemistry of seminiferous tubules epithelial cells on rats

	Cells					
Parameters^1^	Groups^2^	Sertoli	Spermatid	Spermatocyte	Spermatogonia	Leydig
TNF-α	Control	0 (0–1)^b^	0 (0–1)^b^	0 (0–1)^b^	0 (0–1)^b^	0 (0–1)^c^
	VPA	1 (1–2)^a^	1 (1–2)^a^	1 (1–2)^a^	1 (1–2)^a^	2 (2–3)^a^
	CUR	0 (0–1)^b^	0 (0–1)^b^	0 (0–1)^b^	0 (0–1)^b^	1 (0–1)^b^
	VPA+CUR	1 (0–2)^a^	1 (0–2)^a^	1 (0–2)^a^	1 (0–2)^a^	1 (1–2)^b^
IL-6	Control	0 (0–0)^b^	0 (0–0)^b^	0 (0–0)^b^	0 (0–0)^b^	0 (0–1)^b^
	VPA	1 (0–2)^a^	1 (0–2)^a^	1 (0–2)^a^	1 (0–1)^a^	1 (1–2)^a^
	CUR	0 (0–0)^b^	0 (0–0)^b^	0 (0–0)^b^	0 (0–0)^b^	1 (0–1)^a^
	VPA+CUR	0 (0–1)^b^	0 (0–1)^b^	0 (0–1)^b^	0 (0–1)^b^	1 (1–2)^a^
IL-18	Control	0 (0–0)	0 (0–0)	0 (0–0)	0 (0–0)	0 (0–1)^b^
	VPA	0 (0–2)	0 (0–1)	0 (0–1)	0 (0–1)	1 (1–2)^a^
	CUR	0 (0–0)	0 (0–0)	0 (0–0)	0 (0–0)	1 (0–1)^a^
	VPA+CUR	0 (0–1)	0 (0–1)	0 (0–1)	0 (0–1)	1 (1–2)^a^
NF-κB	Control	0 (0–0)	0 (0–0)	0 (0–0)	0 (0–0)	0 (0–1)^b^
	VPA	0 (0–2)	0 (0–1)	0 (0–1)	0 (0–1)	1 (1–2)^a^
	CUR	0 (0–0)	0 (0–0)	0 (0–0)	0 (0–0)	1 (0–1)^b^
	VPA+CUR	0 (0–1)	0 (0–1)	0 (0–1)	0 (0–1)	1 (1–2)^a^

Data are the median value (minimum-maximum). Different superscripts within columns differ (*P*<0.05).

^1^TNF- α: Tumor necrosis factor-alpha; IL-6: Interleukin 6; IL-18: Interleukin 18; NF-κβ: Nuclear factor kappa beta.

^2^Treatments were applied for 14 days. Control = rats given 1 ml of normal saline via oral gavage, VPA = 500 mg/kg valproic acid (dissolved in distilled water) intraperitoneally, CUR = 200 mg/kg curcumin via oral gavage, and VPA+CUR = 500 mg/kg VPA plus 200 mg/kg CUR.

^a ^Significantly different from the VPA group (*P*<0.05).

^b ^Significantly different compared to the control group (*P*<0.05).

CUR: Curcumin; VPA: Valproic acid

## Results

***Biochemistry***

The VPA administration caused a 37% decrease in serum testosterone concentration compared to the control group (Table 1). The CUR treatment restored serum testosterone concentration. Compared to the control group, the VPA administration resulted in 5.32, 9.51, 2.44, and 3.68–fold increases in testicular tissue JAK1, STAT–3, IL–6, and MDA levels, respectively. The CUR treatment completely alleviated testicular tissue JAK1 level and partially alleviated testicular tissue STAT–3, IL–6, and MDA levels.

***Spermatology***

The VPA administration decreased sperm motility and viability by 50% and 52%, respectively (Table 2). The CUR treatment partially improved sperm motility and viability in the VPA group. The median testicular biopsy score for the rats administered with VPA dramatically decreased from 8 to 2. Treatment with CUR partially abolished destruction in testicular cells (Table 2).

***Histopathology***

Histopathological examination of the control group exhibited a normal testis tissue histology (Figure 1A; Table 3). In the VPA group (Figure 1B), there were intense degenerative changes and necrosis in seminiferous tubules. Also, atrophy in some seminiferous tubules was seen. There were irregular views in spermatogenetic cells, the loss of spermatogenic cells, and edema and hyperemia in interstitial areas. The CUR treatment partially reduced the severity of the lesions in the VPA group (Figure 1D; Table 3).

***Immunohistochemistry***

No immune positive reaction for TNF–α, IL–6, IL–18, and NF–κB was observed in the Sertoli cell, germ cells (spermatid, spermatocyte, and spermatogonia), and Leydig cell of the control rats (Table 4; Figure 2). The VPA administration did not trigger the expression of TNF–α and IL–6 in any of the cells but did of IL–18 and NF–κB in only Leydig cells. The CUR treatment was ineffective in altering the expression of TNF–α, IL–6, IL–18, and NF–κB in the Sertoli cell, germ cells (spermatid, spermatocyte, spermatogonia), and Leydig cell of the rats exposed to testicular damage.

## Discussion

Despite considerable side effects, VPA is commonly used in the treatment of epilepsy, an episodic cerebral disorder caused by the increased stimulation of various nerve cells in the brain for various reasons (30). There is increasing concern about the potential effects of VPA on reproductive endocrine function. It decreases the level of testosterone in male patients with epilepsy. Moreover, it causes atrophies of the testes and prostate as well as suppresses spermatogenesis (31). Sperm motility as a reproductive final stage and sperm motility evaluations are an integral part of some reproductive toxicity test guidelines. In the literature, there are studies on the protective properties of various plant-derived and anti-oxidant substances to prevent the damage caused by VPA administration (32-34). This study examined the potential protective effects of CUR on VPA–induced testis damage, where the JAK1/STAT–3/IL–6 pathway and oxidative stress are involved in the pathogenesis.

Mitochondria are required for the energy generation of the cells, and motility and stability of the sperm are necessary for normal mitochondrial function (35), which are compromised by the VPA administration (5). It may also impair cellular mechanisms in different ways that can lead to toxicity by inducing free radical formation and lipid peroxidation. Testicular membranes are structures rich in polyunsaturated fatty acids that are not resistant to oxidative degradation. Lipid peroxidation causes the membranes to lose their functions. MDA levels in tissues and serum increase. The increased MDA level indicates the formation of oxidative stress (36, 37). 

Inflammatory (JAK1, STAT–3, and IL–6) and oxidative status (MDA) markers increased considerably upon the VPA administration, which can be explained by changes in membrane integrity and fatty acid composition and the increased sensitivity of cells to oxidative damage (38). In the study by Ourique *et al*. (39), it was observed that the testicular MDA value increased and sperm motility decreased in oxidative stress-related damage to the testis in rats administered with VPA. Sukhorum and Iamsaard (40) indicated that VPA treatment changed the expression of testicular proteins, which are accountable for spermatogenesis and testosterone generation that lead to infertility. Testicular oxidative stress appears as a common feature in most of the underlying causes of male infertility. It is considered that this situation may benefit the development of anti-oxidant treatments in the relevant cases of hypospermatogenesis (41). With a decrease in sperm motility, damaged sperm cell percentage increased upon the VPA administration, which was associated with decreased testosterone level and destructive histopathology of testicular tissues as well as inflammatory reactions in Leydig cells. In the study by Roste *et al*. (42), testicular atrophy and spermatogenesis arrest were detected in rats administered with VPA at a daily dose of 400 mg/kg. In comparison, no pathology was detected when a daily dose of 200 mg/kg was administered. In this study, rats administered with VPA exhibited degenerative changes, necrosis, and atrophy in the seminal tubules.

The JAK/STAT pathway is a therapeutic target to cure spermatogenesis deterioration (43, 44) because it plays a key role in the occurrence and regulation of the inflammatory response via the transmission of intercellular cytokine signals to the nucleus. Intracellular STAT–3 activation is provided by stimulating IL–6, IL–10, various growth factors, and their receptors (45). This is important for spermatogonial stem cell production and regeneration, which are necessary processes to ensure male fertility. The CUR treatment increased serum testosterone levels and partially restored testicular MDA and JAK1, STAT–3, and IL–6 levels, as well as sperm motility and stability. The CUR treatment partially alleviated the degenerative and necrosis effect of the VPA administration on the seminiferous tubuli, which might result in the loss of spermatogenic cells. The protective effect of CUR could be related to its anti-oxidant property, which alleviates the VPA–induced tissue toxicity (46). Moreover, when used at a higher dose and for a longer time, VPA causes testicular damage by inflammation, which is mediated by NF–κB phosphorylation and increased oxidative stress. The CUR treatments block NF–κB activation through increasing inflammatory stimuli (47). However, the CUR treatment failed to suppress TNF–α, IL–6, IL–18, and NF–κB in Sertoli cells.

## Conclusion

VPA administration exerted destructive effects on testicular tissues. The CUR treatment partially restored the histopathology of testicular damage by restoring antiinflammatory and transcription markers and sperm motility and stability. Further studies should consider testing the CUR treatment at different doses and time periods.

## Declaration

We have not used any AI tools or technologies to prepare this manuscript.
